# Integrative testis transcriptome analysis reveals differentially expressed miRNAs and their mRNA targets during early puberty in Atlantic salmon

**DOI:** 10.1186/s12864-017-4205-5

**Published:** 2017-10-18

**Authors:** K. O. Skaftnesmo, R. B. Edvardsen, T. Furmanek, D. Crespo, E. Andersson, L. Kleppe, G. L. Taranger, J. Bogerd, R. W. Schulz, A. Wargelius

**Affiliations:** 1 Institute of Marine Research, Postboks 1870 Nordnes, 5817 Bergen, Norway; 2 Reproductive Biology group, Department of Biology, Faculty of Science, Utrecht University, Padualaan 8, 3584 CH Utrecht, The Netherlands

**Keywords:** miRNA, Aquaculture, *Salmo salar*, Integrative analysis

## Abstract

**Background:**

Our understanding of the molecular mechanisms implementing pubertal maturation of the testis in vertebrates is incomplete. This topic is relevant in Atlantic salmon aquaculture, since precocious male puberty negatively impacts animal welfare and growth. We hypothesize that certain miRNAs modulate mRNAs relevant for the initiation of puberty. To explore which miRNAs regulate mRNAs during initiation of puberty in salmon, we performed an integrated transcriptome analysis (miRNA and mRNA-seq) of salmon testis at three stages of development: an immature, long-term quiescent stage, a prepubertal stage just before, and a pubertal stage just after the onset of single cell proliferation activity in the testis.

**Results:**

Differentially expressed miRNAs clustered into 5 distinct expression profiles related to the immature, prepubertal and pubertal salmon testis. Potential mRNA targets of these miRNAs were predicted with miRmap and filtered for mRNAs displaying negatively correlated expression patterns. In summary, this analysis revealed miRNAs previously known to be regulated in immature vertebrate testis (*miR-101*, *miR-137, miR-92b, miR-18a, miR-20a*), but also miRNAs first reported here as regulated in the testis (*miR-new289*, *miR-30c*, *miR-724*, *miR-26b, miR-new271, miR-217, miR-216a, miR-135a, miR-new194* and the novel predicted *n268*). By KEGG enrichment analysis, progesterone signaling and cell cycle pathway genes were found regulated by these differentially expressed miRNAs. During the transition into puberty we found differential expression of miRNAs previously associated (*let7a/b/c)*, or newly associated (*miR-15c, miR-2184*, *miR-145* and the novel predicted *n7a and b*) with this stage. KEGG enrichment analysis revealed that mRNAs of the Wnt, Hedgehog and Apelin signaling pathways were potential regulated targets during the transition into puberty. Likewise, several regulated miRNAs in the pubertal stage had earlier been associated (*miR-20a*, *miR-25*, *miR-181a, miR-202*, *let7c/d/a, miR-125b*, *miR-222a/b, miR-190a*) or have now been found connected (*miR-2188*, *miR-144*, *miR-731*, *miR-8157* and the novel *n2*) to the initiation of puberty.

**Conclusions:**

This study has - for the first time - linked testis maturation to specific miRNAs and their inversely correlated expressed targets in Atlantic salmon. The study indicates a broad functional conservation of already known miRNAs and associated pathways involved in the transition into puberty in vertebrates. The analysis also reveals miRNAs not previously associated with testis tissue or its maturation, which calls for further functional studies in the testis.

**Electronic supplementary material:**

The online version of this article (10.1186/s12864-017-4205-5) contains supplementary material, which is available to authorized users.

## Background

In vertebrates, the brain-pituitary system has evolved as the master regulator of pubertal development and adult functioning of the gonads. Fish share many homologies with other vertebrates, also regarding the basic building blocks of the brain-pituitary-gonad (BPG) axis [1–3]. In salmon farming, precocious puberty generates animal welfare problems and inflicts economic damage, since puberty is associated with an increased susceptibility to disease, affects flesh quality, decreases growth and causes hypo-osmoregulatory problems. Precocious puberty is a male-specific problem, since the high energetic requirement of female puberty usually does not allow it to occur until females are harvested [4]. This applied background complements the general scientific interest in understanding the molecular mechanisms implementing testis maturation in animals.

Several proteins in fish testis regulate the initial steps of spermatogenesis such as Amh, Gsdf, Igf3 and Insl3 in eel [5, 6], rainbow trout [7] and zebrafish [8–10]. A few transcriptome studies in fish have reported changes in testicular mRNA levels and provided information on the potential involvement of genes and pathways regulating pubertal testis maturation [11–14]. In addition to mRNAs, miRNAs impact every regulatory pathway in animals [15], while there is limited information on the potential roles of miRNAs in testis maturation in fish [16]. miRNAs are short (~19–23 nucleotides) palindromic noncoding RNA species that specifically target and bind 3′-UTR regions of mRNAs, resulting in post-transcriptional silencing via accelerated mRNA decay and translational repression in a mechanism mediated by Argonaute family members [17]. Hence, miRNAs regulate the abundance and usage of expressed mRNAs. Also a given miRNA can target more than one mRNA, since different mRNAs can share the region complementary to the miRNA’s seed region [18].

The significance of miRNAs for testicular function has been demonstrated in several studies and work in mice has identified miRNA processing enzymes in testis as essential [16, 19–22]. Additional studies have also established functions for specific miRNAs in regulating spermatogenesis, including the *miR-17-92* family [23], the testis-enriched and sexually-dimorphic *miR-140* [24], the retinoic acid (RA)-induced *miR-146*, and the clustered *miR-221* and *miR-222* [25–27]. Several studies described miRNAs in commercial fish species such as yellow catfish [28], halibut [29, 30] and Atlantic salmon [31–33]. The latter studies described the miRNA repertoire in Atlantic salmon tissues, but did not investigate miRNA expression in testis. The first study dealing with the testicular miRNA repertoire in salmonids examined immature and fully mature testis of rainbow trout [34]. However, comparing the start and end points of a developmental process stretching over several months provides a limited amount of specific information on the potential functional context of changes in miRNA expression.

In view of our interest in early stages of spermatogenesis and testis maturation, we concentrate on miRNAs of potential relevance for initiating puberty. To identify suitable testis tissue samples, we monitored a group of 2-year-old salmon for 10 months and defined three groups based on morphological, physiological, and molecular parameters: immature males, males a few weeks before (prepubertal), and males shortly after the initiation of testis maturation (pubertal). Since the main known function of miRNAs is to regulate the use of expressed mRNAs, we decided to analyze, by RNA sequencing, both mRNA and miRNA repertoires in the same set of samples. The potential interaction of miRNAs with target mRNAs was then investigated, by examining the expression correlation of predicted targets of differentially expressed miRNAs. With this integrated approach mRNA targets predicted by the miRmap algorithm [35] were refined to targets showing an inversely correlated expression to their respective targeting miRNA. Utilizing this approach, we have identified sets of testicular miRNAs targeting pathways known to be involved in preventing or promoting differentiation processes in the vertebrate testis in addition to in this respect potential new miRNA targets.

## Results

### Sample characterization

**Fig. 1:**
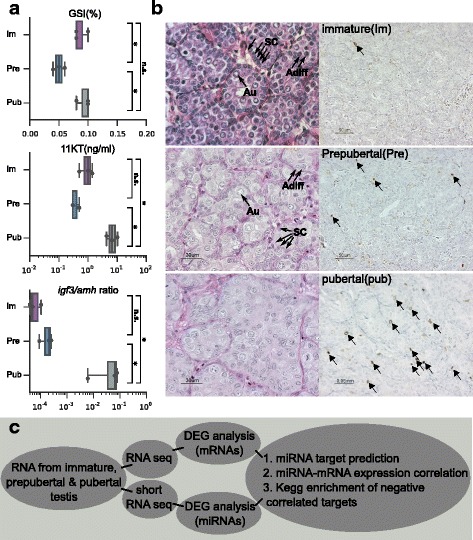
Description of samples and analysis procedure. (**a**) Gonadosomatic index (GSI, %; upper), 11-ketotestosterone levels (11KT, ng/ml; center) and the ratio of gene expression for igf3/amh (lower) in testis samples used for miRNA and mRNA-seq analysis. Statistical significant differences are marked with asterisk (*: p < =0.05, n.s.: non significant, according to Wilcoxon rank-sum statistic). (**b**) Representative histology (hematoxylin-eosin combined with periodic acid according to Schiff; HE/PAS; left column) and proliferation activity (immunocytochemical detection of phosphorylated histone H3; pH 3; right column, marked with triangles) in immature (Im), prepubertal (Pre) and pubertal (Pub) testis samples. Abbreviations: Au = type A undifferentiated spermatogonia, and Adiff = type A differentiating spermatogonia, SC = Sertoli Cells (**c**) Illustration of experimental setup and analysis pipeline used in this paper

To identify cues controlling molecular pathways associated with puberty in Atlantic salmon, we characterized differentially expressed miRNAs and mRNAs in samples from immature, prepubertal and pubertal testis. To select suitable testis tissue samples, we investigated several maturity associated parameters, including the gonado-somatic index (GSI), plasma levels of 11-ketotestosterone (11-KT) and the relative expression levels of *amh* and *igf3* in testis (Fig. 1A). These measurements were complimented by histological analysis of germ cell development and immunohistochemical analysis of testicular proliferation activity. These combined analyses allowed selecting males for assignment of their testis tissue samples into three groups; an immature stage, a prepubertal stage showing limited proliferation activity and a pubertal stage showing elevated single cell proliferation activity of both, spermatogonia and Sertoli cells (Fig. 1B). Based on this grouping, we decided to in depth characterize differentially expressed miRNAs and their inversely correlated targets in Atlantic salmon testis (Fig. 1C). A more detailed description of the tissue sample donors can be found in Additional file 1.

### Integrated expression analysis

**Fig. 2:**
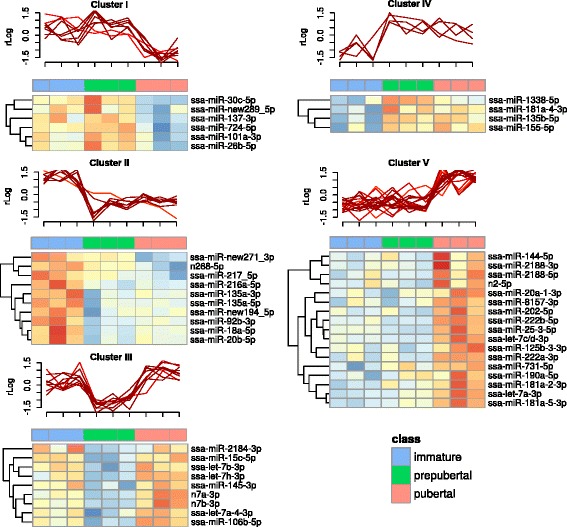
Hierarchical clusters of miRNAs that are differentially expressed in salmon testis. Heatmap displaying 5 hierarchical clusters of miRNAs that are differentially expressed in salmon testis during onset of puberty (values are depicted as DESeq2 normalized rlog expression values (log2 scale). On the x-axis, the type of sample is illustrated with color codes (immature-blue, prepubertal-green, pubertal-pink). Above the heatmaps, expression profiles are depicted. The k-means profile plots highlighted in light-red correspond to less similar profiles**.** On the y-axis DESeq2 normalized rlog values are plotted (log2 scale)

**Fig. 3:**
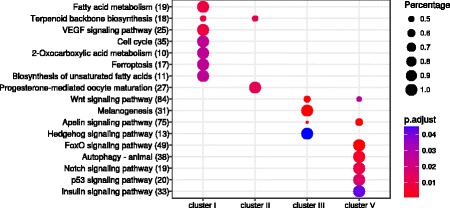
KEGG pathway enrichment of target mRNAs associated with miRNA clusters. Functional enrichment of the 20% top scoring miRmap target predictions for each miRNA where an association were supported by a negatively correlated mRNA expression profile (Pearson correlation < = − 0,5). Dots represent KEGG term enrichment where the color coding indicates the magnitude of the adjusted *p*-values (p.adj). Adjusted p-values ranges from 0.0415581719069 to 5.5547769425e-08, where a red color indicates a lower p-value and a blue color represents a higher p-value). The sizes of the dots represent the percentage of each row (KEGG category). On the y-axis in parentheses after the KEGG term is the number of genes found in the pathway. Cluster IV is not represented as target genes for this cluster of miRNAs produced no KEGG term enrichment

Differential miRNA expression analysis revealed five expression clusters (I-V, Fig. 2). We analyzed correlations between expression profiles from the miRNA expression dataset with expression profiles of predicted and differentially expressed targets from an mRNA expression dataset derived from the same samples. Differentially expressed mRNAs are provided in Additional file 2. In a similar approach as undertaken by others [36–38], we focused on analyzing inverse correlated miRNA -mRNA target pairs with the assumption that high confident miRNA-mRNA targeting pairs display a negative correlation as the result of mRNA deadenylation and destabilization which alongside with translational repression is considered the mechanism of miRNA repression [39]. This analysis revealed that the 20% best scoring predicted targets for each miRNA (Additional file 2), when refined by considering only negatively correlated target mRNAs (Pearson correlation <= − 0.5), had specific enrichment of KEGG pathways (Fig. 3) associated to each miRNA expression cluster (Fig. 2, Additional file 2).

**Fig. 4:**
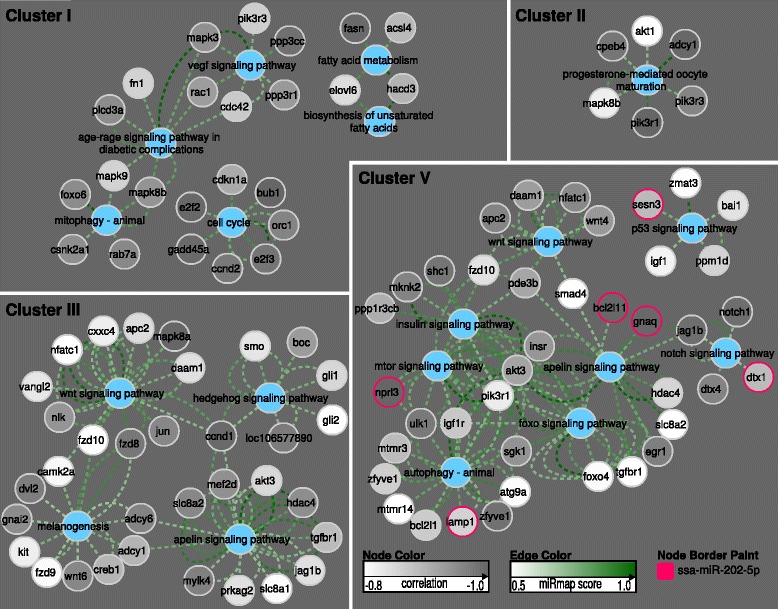
Target genes in KEGG pathways. Single genes/pathways significantly associated with miRNA clusters. A Cytoscape network rendering of enriched KEGG pathways and their miRNA targeted gene members. The color of the nodes is assigned according to the strength of the negative Pearson correlation of expression profiles of its targeting miRNA (Pearson correlation −1 to −0.8, dark grey to white). The dotted lines connecting the nodes (edges) are colored from light green to green according to the corresponding strength of the miRNA target prediction (miRmap target prediction score, white to green). Red rings around nodes in cluster V are depicting genes predicted to be regulated by *miR-202*. Raw data can be manually inspected in Additional file 2 (correlation and miRmap scores). Cluster IV is not represented as target genes for this cluster of miRNAs produced no KEGG term enrichment

Cluster I contained six miRNAs (*miR-30c*, *miR-new-289*, *miR-137*, *miR-724*, *miR-101a* and *miR-26b*) that had a higher expression in the immature and prepubertal stages than in the pubertal stage. By KEGG enrichment, cluster I displayed potential target mRNAs representing genes involved in cell cycle and metabolic pathways (Figs 3, 4). Of the genes associated to cell cycle, several genes were related to mitosis (*cdc27*, *cdc23*), both meiosis and mitosis (*fzr1*, *bub1b*) or meiosis (*cdkn1a*) [40–42]. Interestingly, *Cdkn1a* has previously also been associated with Fsh-induced stimulation of spermatogonial differentiation in zebrafish [12]. Other genes possibly related to the maturing gonad are *wee1-b* and *e2f2* which encode proteins associated with stem cell renewal, cell proliferation and controlled proliferation through apoptosis in other animals [43, 44]. High scoring mRNA target predictions of the cell cycle pathway were all predicted as targets of either *miR-724*, *miR-137*, *miR-30* or *miR-101a*. Two of these miRNAs have been associated with testis function previously, since *miR-101* and *miR-137* were negatively regulated in response to RA-induced spermatogenesis in dogs [27].

Cluster II contained miRNAs with a higher expression in the immature relative to the prepubertal and pubertal stages. This cluster contained ten miRNAs (*miR-new-271*, *miR-217*, *miR216a*, *miR-135a*, *miR-new-194*, *miR-92b, miR-18a*, *miR-20b* and the novel predicted miRNA *n268*). KEGG enrichment analysis revealed possible target genes belonging to the progesterone-mediated oocyte maturation pathway (Figs 3 and 4). Some of the miRNAs found in this cluster have previously been associated with a function in the testis, like *miR-18a* and *miR-92b* which belongs to the *miR-17-92* cluster that is essential for spermatogenesis in mice [23]. *miR-92b* is also highly abundant in zebrafish gonads and spermatozoa [45]. The other miRNAs found in cluster II, *miR-new271*, *miR-217*, *miR-216a* and *miR-135a* have, however, not been linked yet to a function in the testis.

Cluster III included nine miRNAs (*miR-2184*, *miR-15c*, *let-7b/h/a*, *miR-145*, *miR-106b,* and the novel predicted *n7a and b*) that displayed a drop in expression during the prepubertal stage. Their reduced expression during this transitional stage suggests that suppression of targeted mRNAs is alleviated at this stage, potentially allowing entry into maturation. The KEGG enrichment of miRNA targets for cluster III involved Wnt, Hedgehog, Apelin and Melanogenic signaling (Figs 3 and 4). High scoring targets in these pathways displayed target relations to the following miRNAs; the novel predicted miRNAs *n7a* and *n7b, let7a/b/h*, *miR-106b* and *miR-2184*. *let-7a* and *let-*7b have also been demonstrated to peak in expression in rat testis during puberty, implicating a conserved modulating role for these miRNAs in vertebrate puberty [46]. *miR-106b* has been shown previously to be downregulated during RA-induced spermatogonial differentiation in mouse [23]. *miR-2184* has been found previously in medaka testis, but has not been linked to puberty so far [47].

Cluster IV contained only 4 miRNAs (*miR-1338*, *miR-181a*, *miR-135b* and *miR-155*) that displayed a lower expression in immature but higher expression in the prepubertal and pubertal testis. While no predicted enriched pathways were retrieved for this cluster, its expression profile is compatible with the notion that miRNAs of cluster IV may have a role in suppressing targets involved in keeping the testes in an immature state.

Cluster V consisted of a relatively large group of 17 miRNAs (*miR-144*, *miR-2188*, *miR-20a*, *miR-8157*, *miR-202*, *miR-222b*, *miR-25*, *let7c/d/a, miR-125b*, *miR-222a*, *miR-731*, *miR-190a, miR-181a* and the novel *n2*), characterized by a lower expression in immature and prepubertal testis. Among potentially targeted pathways regulated by miRNAs in this cluster, we found mRNAs associated with the interconnected Apelin, Wnt, FoxO, insulin, mTor, Notch and Autophagy signaling pathways (Fig. 4, listed in Additional file 2). Key miRNAs including the novel predicted miRNA *n2*, *let7a and c*, *miR-125b*, *miR-144*, *miR-181*, *miR-190a*, *miR-202*, *miR-20a*, *miR-2188*, *miR-731* and *miR-8157* were supported by both a high prediction score and a negative correlation to targets of these pathways (Fig. 4, Additional file 2). Interestingly, *miR-125b, let7a* and *c* have been found expressed in dog testis [27]. *miR-202* is expressed in Sertoli cells and a knockout model in mice has demonstrated that this miRNA as essential for spermatogonial stem cell survival [48]. *miR-202* is also highly abundant in the testis of trout, representing 20% of all microRNAs found in testis tissue [34], further underscoring the significance of this miRNA. We observed in our dataset that *miR-202* is targeting many genes in the above-mentioned pathways including *dtx1*, *gnaq*, *bcl2l11* and *nprl3* (highlighted with red rings in Fig. 4) in addition to *tgfbr-1* (Additional file 2). *Tgfbr-1* and *smad4* are key signaling transmitters of the Tgf-β superfamily ligands such as the testis maturation inhibiting factor Amh [1, 49, 50], and are in our data predicted as potential targets of *miR-181a* and *miR-190a,* respectively (Additional file 2, cluster V); *miR-190a* was also regulated during bovine oocyte maturation [51]. Jag1 and Notch1 are single pass transmembrane receptors in the Notch signaling pathway that in our data are predicted targets of *miR-144* and *let7c,* respectively (Fig. 4 and Additional file 2). Additionally, we observed that *igf1* and *igfr1,* in mice identified as stimulating Sertoli cell proliferation [52], are potential targets of *let7a and c, miR-2188*, *miR-202* and *miR-125b* (Additional file 2, cluster V). Two miRNAs in cluster V, *miR-25* and *miR-181a,* were abundant in zebrafish spermatozoa [45], while *miR-2188* is expressed in halibut gonads but has not been associated with testis maturation yet [30]. *miR-20a*, a sexually dimorphic miRNA, is a possible target of DMRT-1 in the testis of Nile tilapia [53]. In addition to miRNAs that previous work has linked to testis maturation, we found miRNAs in cluster V that have not been reported so far as linked to testis maturation, such as *miR-731*, *miR-8157* and the novel predicted *n2*.

## Discussion

While a previous study has described differences in miRNA repertoires between immature and fully mature testis in a related species, the rainbow trout [34], we were interested in finding triggers of maturation through monitoring testicular RNA repertoires in a narrower time window around the initiation of testis maturation and contrasting this with immature testis months away from the potential start of puberty. For the selection of testis tissues, our criteria included the measurement of several parameters involved in determining maturity such as plasma 11KT/testosterone levels, gonadosomatic index (GSI) and testicular expression of *igf3* and *amh* in conjunction with histological and immunohistochemical evaluation of proliferation. Recombinant zebrafish Igf3 stimulated [9] while recombinant zebrafish Amh inhibited spermatogonial differentiation [10] and androgen treatment of immature salmon promoted spermatogonial differentiation associated with elevated *igf3* but reduced *amh* testicular transcript levels [54]. The ratio of these transcripts was therefore used as a measure of maturity. These combined analyses allowed selecting males for assignment of their testis tissue samples into three groups; an immature, quiescent stage months before the potential start of maturation; a prepubertal stage showing little proliferation activity; and a pubertal stage showing elevated single cell proliferation activity of both, spermatogonia and Sertoli cells (Fig. 1B), just after the start of seasonal testis growth. An integrative miRNA-mRNA transcriptome analysis of these samples revealed specific miRNA expression patterns as well as KEGG pathway enrichment of negatively correlated and high confidence predicted mRNA targets of these miRNAs.

The miRNAs belonging to expression cluster I showed a higher expression in the immature and prepubertal stages. We suspect therefore that these miRNAs might mediate suppression of puberty-inducing mRNAs. By KEGG enrichment analysis, we found the predicted targets of miRNAs in this cluster to be enriched for mRNAs that code for proteins involved in the cell cycle pathway. This observation seems consistent with a down-regulation of these mRNAs during a stage were the gonad remains quiescent with a low cell division and metabolic activity.

miRNAs in cluster II are preferentially expressed in the immature stage and might thus also act to suppress maturation inducing mRNA targets. As these miRNAs displayed a sharp decrease in expression past the immature stage they are, however, more likely to target a subset of mRNAs that are released from miRNA-mediated inhibition earlier than those targeted by cluster I. The mRNAs enriched in the progesterone-mediated oocyte maturation pathway might be relevant in this respect, since signaling by the progestin 17α,20β-dihydroxy-4-pregnen-3-one (DHP) through this pathway is an important step in stimulating the spermatogonial phase in fish spermatogenesis [55, 56]. In adult zebrafish, DHP treatment doubled the testis weight, increased spermatogonial proliferation/differentiation and affected expression of several growth factor genes [57]. Targeting of specific transcripts in the progesterone pathway by either *miR-135a*, *miR-92*, *n268* or *miR-20b* as observed in our data (Fig. 4, Additional file 2) might thus act to prevent the gonad from entering puberty.

miRNAs in cluster III displayed a dip in expression in the prepubertal stage and we therefore speculate that these miRNAs target mRNAs that need to be transiently derepressed in order to allow entry into puberty. The mRNA targets of these miRNAs were associated with the Wnt, Hedgehog and the Apelin signaling pathways. Interestingly, the Apelin pathway is noted to contain also members of the Tgf-β pathway, which in conjunction with both the Wnt and Hedgehog signaling pathways had been associated with proliferation activity in and growth of the zebrafish testis [12]. Studies on rat testis have additionally shown that Hedgehog signaling promotes the survival of germ cells and can be suppressed by FSH, a key endocrine regulator of germ cell differentiation [58]. By targeting *gli1* (*n7a*), *gli2 (let-7 h)* and *smo* (*let7b, miR-106b*), all being downstream mediators of Hedgehog signaling, these miRNAs might modulate the Hedgehog pathway during entry into puberty (Additional file 2: Table S1).

Cluster IV and V contained miRNAs that had a higher expression towards the onset of maturation. These miRNAs we thus suspect to be involved in suppressing processes that would prevent the onset of testis maturation. We found no enriched pathways for targets of miRNAs expressed in cluster IV. Considering the relatively few miRNAs in this cluster it is possible that their cumulative targets were too few to result in any pathway enrichment. Cluster V, however, was enriched for target mRNAs associated with the interconnected Apelin, Wnt, FoxO, insulin, mTOR, Notch and Autophagy signaling pathways (Fig. 4, Additional file 2). *Smad4* and *tgfbr-1* which both are central to signaling by the Tgfβ superfamily ligands such as the testis maturation inhibiting hormone Amh [1, 49, 50], were predicted targets of *miR-190a* and *miR-181a* respectively. *Tgfbr-1* was additionally found targeted by *miR-202* (Additional file 2), a miRNA that recently has been found to be essential for spermatogonial stem cell survival [48]. The targets of *miR-202* remain largely undefined. Besides *tgfbr1,* only *bcl2l11,* a pro-apoptotic protein, could be linked to a function in spermatogenesis [59]. For *nprl3* and *dtx1*, negative regulators of mTOR and Notch signaling, respectively, no function has been reported previously in the testis although both pathways have been implicated in regulation of maturation [12, 60]. While *gnaq* is expressed in the sheep testis [61], its function is unknown.

We observe also a possible involvement of miRNAs in modulating insulin signaling, by targeting *igf1* and *igfr1*. It is possible thus that these miRNAs in salmon have an important role in modulating Igf action during puberty. Interestingly, an experimental support for a *let-7/igf1* target regulation in gonads has been determined in mice [62]. The Notch pathway is also relevant as it constitutes an evolutionary conserved mechanism mediating cell-cell communication between single-pass transmembrane receptors including, in our study, the targeted *Jag1b* and *Notch1*. Interestingly, roles for Notch signaling in fetal Leydig cells and in the communication between Sertoli cells and spermatogonia have been described, and in both mice and zebrafish, Notch signaling has been associated with initial or early stages of testis maturation [12, 63, 64]. Our findings therefore suggest that there might exist a previously unrecognized role for miRNAs in modulating the Notch pathway affecting early stages of testis maturation.

## Conclusion

In this study we have identified miRNAs and their associated suppressed mRNAs/pathways, which could function either as activators or repressors of maturation. We identified both known and unknown miRNAs that potentially regulate pathways involved in the start of testis maturation.

Since a large proportion of the identified miRNAs has been associated with testis maturation in other vertebrates previously, conserved roles for these miRNAs seem possible (*miR-101*, *miR-137, miR-18a, let7a/b/c*, *miR-92b, miR-20a*, *miR-25*, *miR-181a, miR-202*, *let7c/d/a, miR-125b, miR-190a* and *miR-222a/b*). On the other hand, we identified miRNAs not previously reported in the context of testis maturation (*miR-new289*, *miR-15c, miR-20b, miR-30c*, *miR-724*, *miR-26b, miR-new271, miR-217, miR-216a, miR-135a, miR-144*, *miR-145, miR-new194, miR-2184*, *miR-2188*, *miR-731*, *miR-8157* and the novel predicted miRNAs *n2, n7a and n7b,*). We have thus identified testicular miRNAs and their associated mRNAs, differentially expressed during the initiation of pubertal spermatogenesis. In comparison to previous papers reporting miRNAs associated to reproduction in fish, this paper utilizes an integrated approach considering differential expression of miRNAs and their predicted targeted mRNAs from the same tissue samples that were carefully selected for their different stages of development and activity, without showing important changes in the cellular composition of the tissue. However, the relatively low sample size may represent an uncertainty in this study. Further studies including functional reporter assays are therefore required to reach firm conclusions on target relations. Nevertheless, the presented data within this study provides a valuable resource and a starting point for future investigation of these matters.

## Methods

### Fish and sampling

The fish used in this study were Atlantic salmon postsmolts of defined genetic background (Aquagen strain). They were siblings or closely related, all from the same Aquagen strain. The fish were hatched and reared in freshwater indoor facilities at the Institute of Marine Research, the Matre Aquaculture Research Station, Matredal (61°N), Norway, and transferred to sea cages after smoltification at 18 months of age, until January (6 months in sea water). Males used for the prepubertal and pubertal samples were all sampled in January while the immature fish were sampled in June. Fish were netted from the cage, immediately anesthetized with 6 ppt metomidate (Syndel, Victoria,B.C.) and weighed (total body weight). Blood (5 ml) was collected in heparinized syringes from the caudal vein. Subsequently, the fish were killed by cutting the medulla oblongata, after which gonad tissues were excised. Gonads were weighed for gonado-somatic index (GSI) determination (GSI = gonad weight (g) *100/total body weight (g)) and central transversal pieces of testes tissue were cut with a scalpel blade. One piece of tissue was fixed in Bouins fixative for histological analysis, and another piece was immediately frozen in liquid nitrogen. Immunocytochemical detection of phosphorylated histone H3 was done according to established protocols [54]. Plasma samples were prepared according to Melo et al., 2015 [54] and quantification of 11-KT was performed by a radio-immuno assay [65]. In total 66 male fish were sampled, 3 were classified as immature, 3 as prepubertal and 3 as pubertal. A detailed description of samples used in the study can be found in Additional file 1.

### RNA extraction

Total RNA including the small RNA fraction was extracted from 20 mg of testis tissue samples, stored in liquid nitrogen, using the miRNeasy kit (Qiagen), according to the manufacturer’s instructions. RNA samples were subsequently treated with TurboDNAse (Ambion) to remove any contaminating genomic DNA. RNA was analysed with a Agilent RNA 6000 Nano Kit (Agilent) and no traces of genomic DNA could be detected.

### mRNA and miRNA deep sequencing

Preparation of cDNA for sequencing on the Illumina Hiseq2000 instrument was performed by the Norwegian Sequencing Centre using the Illumina TrueSeq small RNA library prep kit for miRNA and the TrueSeq RNA library prep kit for mRNA. mRNA and miRNA samples(9 samples each) were multiplexed and sequenced (100 bp read length) on 4 Illumina HiSeq2000 lanes. For mRNA paired end sequencing was utilized, whereas for miRNAs single end sequencing was used. Raw sequence files were de-multiplexed by the Norwegian Sequencing Centre and provided to us as FASTQ files together with an initial FASTQC quality report [66]. The microRNA data and RNA-seq have been deposited to SRA, bioproject numbers PRJNA383545 and PRJNA380580, respectively.

### Fastq filtering and data analysis by miRDeep2

miRNA reference files were downloaded from miRbase v21 [67] and processed by conversion to DNA sequences and removal of sequences containing sequence bases other than u,t,a,g,c. Fastq files trimmed by FastqMcf [68] were then analyzed using the miRdeep2 Perl scripts [69]. Filtered reads were mapped against a bowtie index of the Atlantic salmon genome (ICSASG_v2) using the miRdeep2 mapper.pl command. Next, in order to discover and quantify known and potential new miRNAs, the miRDeep2.pl command was executed, with miRNA reference files containing miRBase deposited miRNA sequences as well as previously identified novel salmon miRNA sequences [32, 33]. The output of miRDeep2 were then cured and annotated by performing directional blast searches against miRBase v.21. Duplicate entries were removed, and repeat elements were removed by tandem repeats finder [70]. Novel miRNAs previously found by Bekaert et al. [33] are preceded by ssa-miR-new or ssa-miR-nov. Unannotated potentially novel miRNAs detected by the miRdeep2 pipeline were given a running number preceded by an n, indicating that these are potentially novel miRNAs (Additional file 3).

### Gene expression analysis

The gene expression table produced by miRdeep2 was filtered to remove miRNA entries where the maximum depth of coverage among the libraries was less than 10 reads per sample and thereafter normalized by the Rlog function in the DESeq2 package [71]. The differentially expressed miRNAs reaching statistical significance, as determined by Benjamini & Hochberg multiple test correction in DESeq2 (p < =0.05), were extracted and subjected to hierarchical and k-means clustering using the R-packages pheatmap and Mfuzz respectively [72, 73]. The expression levels of differentially expressed miRNAs can be found in Additional file 4. Paired end sequences from the mRNA seq were mapped with Bowtie2 [74] against the the Atlantic salmon genome (ICSASG_v2) and a raw count table was extracted with the use of SAMtools [75]. Entries where the maximum depth of coverage among the libraries was less than 10 reads per sample were discarded and differentially expressed genes (DEG) were computed with DESeq2, retrieving only DEG with a an absolute log2fold change > = 0.5 and an adjusted *p*-value <=0.05. Mapping statistics and gene expression tables are provided as supplementary tables (Additional files 5 and 2).

### Prediction of miRNA targets and pathway analysis

3′-UTR’s sequences for salmon mRNAs were extracted as the sequence spanning from CDS stop to the end of transcripts using a genbank flat file hosted at ncbi (GCF_000233375.1_ICSASG_v2_rna.gbff.gz). In cases where there were multiple annotations for a transcript, the longest 3′-UTR was retained. Sequences for each 3′-UTR was then compiled to a multi fasta file. Based on this salmon 3′-UTR reference multi fasta file, targets for differentially expressed miRNAs were predicted with miRMap [35]. Expression correlation between miRNAs and miRNA targets was calculated based on expression profiles from miRNAs as well as mRNAs from the same samples: An expression profile for averaged measurements across stages for each miRNA & mRNA was made. Then using the python pandas.expanding_corr function, a pairwise Pearson correlation coefficient (1: perfectly correlated, −1: anticorrelated) was calculated for these expression profiles. For downstream pathway analysis only mRNAs supported by a predicted miRNA-mRNA relation (based on the 20% top scoring miRmap target predictions for each miRNA) and with an anti-correlated expression (Pearson < −0.5), were used as input for KEGG analyses using the R ClusterProfiler package [76]. Target predictions and associated pathways were then visualized in Cytoscape [77]. In order to prevent excessive cluttering of the network, only a subset of these target relations consisting of the top 50% scoring miRNA predictions for each pathway and with a negative expression correlation less than −0,8 were visualized. Nodes of the network (genes) were colored according to the strength of the negative correlation with their respective targeting miRNAs (Fig. 4).

### Quantitative PCR analysis

We cross-validated the differential expression analysis with miRNA specific rt.-qPCRs using primers designed with miRprimer [78]*.* Conversion of RNA to cDNA was performed following the poly(A) polymerase (PAP) cDNA synthesis protocol, where non-polyadenylated RNA species like miRNAs are converted to a polyadenylated RNA intermediate by the use of *E. coli* poly(A) polymerase and primed with an anchored polydT primer containing an anchor site for a miRNA specific reverse primer in its 5′-end. The PAP cDNA synthesis and primer design was performed as described previously [78, 79]. The cDNA was diluted 1 to 20 and 0.5 μl of this diluted cDNA was utilized in a 6 μl qPCR reaction with 3 μl Fast SYBR green master mix (Applied Biosystems) and 3 μl of a 0.5 μM miRNA specific primer mix (Additional file 6). For about half of the miRNAs we could confirm an excellent correlation (Pearson correlation > =0.98) between the deep-sequencing and RT-qPCR assays, and the majority of assays displayed a Pearson correlation greater than 0.8. For a few miRNAs, the RT-qPCR assays had Pearson correlation coefficients <0.8 (*miR-135a-3p*: 0.76, *miR-135b-5p*: 0.72, *miR-181a-4-3p*: 0.56, *miR-202-5p*: 0.63, *miR-137-3p*: 0.64). It is likely that the primer design influences the success rate of a RT-qPCR assay and with miRNAs this has been reported to be particularly challenging due to the constrained positioning of the primers [78, 79]. Testicular transcript levels of *igf3*/*amh* were quantified as described previously [54].

## Additional files

Additional file 1: A detailed description of samples used in the study. (DOCX 6 kb)

Additional file 2: Table S1. A complete overview of miRNAs obtained from cluster I-V,their high scoring predicted negatively correlated targets and KEGG enriched associated pathways. In a separate tab the 20% highest scoring targets per miRNA (as assessed by miRmap score) not filtered by correlation is provided. Differential expressed mRNAs of pairwise comparisons between the immature, prepubertal and pubertal testis stages are also provided together with their respective Rlog normalized expression values in separate tabs. (XLSX 6839 kb)

Additional file 3: Table S2. miRNA reference file (sequences of mature and precursor miRNAs identified in this study). (XLSX 96 kb)

Additional file 4: Figure S1. Volcano plots of pairwise comparisons displaying differentially expressed miRNAs, their Log2fold changes and *p*-values. (PDF 242 kb)

Additional file 5: Table S3. Mapping statistics of mRNA and miRNA mapping. (XLSX 7 kb)

Additional file 6: Table S4. Primers. (PDF 39 kb)

## Abbreviations

11-KT: 11-ketotestosterone
3′-UTR: Three prime untranslated region
BPG: Brain-pituitary-gonad
CDS: Coding sequence
DHP: 17α,20β-dihydroxy-4-pregnen-3-one.
GSI: Gonado-somatic index
KEGG: Kyoto Encyclopedia of Genes and Genomes
miRNA: microRNA,
RA: Retinoic acid
