# Supplementary material for: Integrative testis transcriptome analysis reveals differentially expressed miRNAs and their mRNA targets during early puberty in Atlantic salmon
Source: BMC Genomics. 2017 Oct 18;18:801. doi: 10.1186/s12864-017-4205-5 (PMC5648517; doi:10.1186/s12864-017-4205-5)
Supplement: Supplementary file 1 — A detailed description of samples used in the study. (DOCX 6 kb) [file 12864_2017_4205_MOESM1_ESM.docx]

**Supplementary description 1**

The immature group was characterized by low 11-KT levels that did not show a noteworthy increase in all 5 samples collected over the last 6 months prior to sampling in June. While it cannot be excluded that these males may have matured following the next winter solstice, the potential start of puberty was at least 5-6 months ahead, i.e. data from this group are considered representing not only an immature but a long-term quiescent testis tissue. These males showed a low *igf3*/*amh* ratio. Histological analysis showed type A spermatogonia as the furthest developed germ cell type. Importantly, germ and Sertoli cells showed little proliferation activity (Fig. 1B, Im). Similar to the situation in zebrafish [1], there are two types of A spermatogonia in the Atlantic salmon: the more undifferentiated type shows a large nucleus with little heterochromatin (type A undifferentiated, A_u_) and the more differentiated type A differentiating (A_diff_), showing a smaller nucleus with more heterochromatin. Often, several Sertoli cell nuclei were aligned in a single row oriented towards the tubular wall; many of these Sertoli cells did not appear to be in contact with germ cells.

The prepubertal group was composed of males sampled in January 2009. Plasma androgen levels and the stage of germ cell development (type A spermatogonia) were not different while the proliferation activity seemed slightly higher than in the immature group. However, sampling of these males occurred close to the start of puberty, considering that practically all males (96%; unpublished observation) had started to mature a few weeks later until the end of February. The *igf3*/*amh* transcript ratio showed a first sign of (yet not significant) increase compared to the immature group. Histologically, we noted that several Sertoli cell nuclei also appeared between the spermatogonial nuclei and not predominantly close to the tubular wall (Fig. 1B). Taken together, these males are considered representing a developmental stage just before entering puberty.

Pubertal males were also sampled in January, still showed low GSI values and type A spermatogonia as the furthest developed germ cell type (Fig. 1B). It is important to emphasize that GSI levels and cellular composition were rather similar between the three groups, increasing the likelihood that differences in miRNA/mRNA expression reflect regulatory processes. Yet, all other parameters differed clearly: the *igf3*/*amh* transcript ratio increased nearly 300-fold (Fig. 1A), based on both, increasing *igf3* and decreasing *amh* transcript levels. Also, the plasma androgen levels had increased clearly. Larger spermatogenic tubuli contained more type A spermatogonia and Sertoli cells (Fig. 1B); the nuclei of the latter were often found in groups arranged among single germ cells. Proliferation analysis demonstrated an activation of single cell proliferation (Fig. 1B) of both type A spermatogonia and Sertoli cells (Fig. 1B). Samples showing clonal proliferation of smaller or larger groups of germ cells, representing the expansion of differentiating spermatogonial clones [2], were excluded from the study, since they were considered as being too far progressed and often already contained type B spermatogonia. Hence, data from this group was considered representing the earliest stage of pubertal activation of the testis, not yet showing a shift in the cellular composition of the testis, despite the increased number of cells, reflecting activation of single cell proliferation.

**References**

1. Leal MC, Cardoso ER, Nóbrega RH, Batlouni SR, Bogerd J, França LR, et al. Histological and stereological evaluation of zebrafish (Danio rerio) spermatogenesis with an emphasis on spermatogonial generations. Biol. Reprod. 2009;81:177–87.

2. Schulz RW, de França LR, Lareyre J-J, Le Gac F, LeGac F, Chiarini-Garcia H, et al. Spermatogenesis in fish. Gen. Comp. Endocrinol. 2010;165:390–411.
