# Supplementary figures and images for: Integrative testis transcriptome analysis reveals differentially expressed miRNAs and their mRNA targets during early puberty in Atlantic salmon
Source: BMC Genomics. 2017 Oct 18;18:801. doi: 10.1186/s12864-017-4205-5 (PMC5648517; doi:10.1186/s12864-017-4205-5)

Prepubertal vs Immature

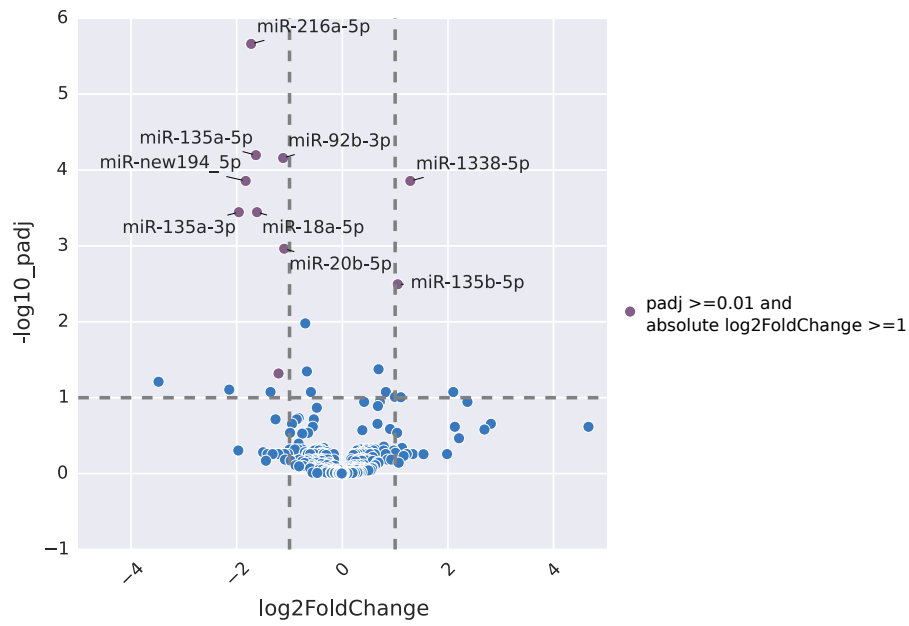

Pubertal vs Prepubertal

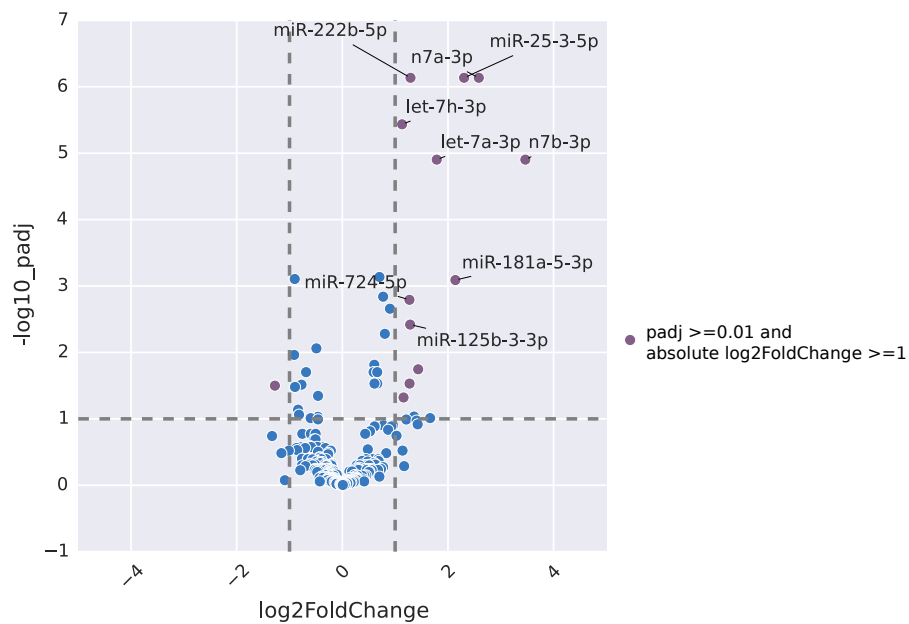

Pubertal vs Immature

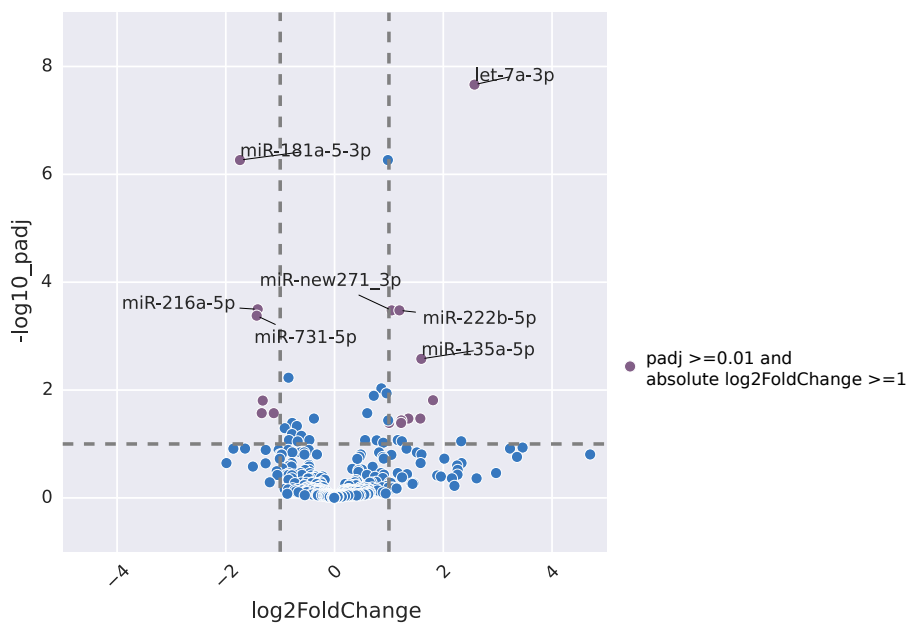

Supplement: Supplementary file 4 — Volcano plots of pairwise comparisons displaying differentially expressed miRNAs, their Log2fold changes and p-values. (PDF 242 kb) [file 12864_2017_4205_MOESM4_ESM.pdf]
