# Supplementary material for: Integrative testis transcriptome analysis reveals differentially expressed miRNAs and their mRNA targets during early puberty in Atlantic salmon
Source: BMC Genomics. 2017 Oct 18;18:801. doi: 10.1186/s12864-017-4205-5 (PMC5648517; doi:10.1186/s12864-017-4205-5)
Supplement: Supplementary file 6 — Primers. (PDF 39 kb) [file 12864_2017_4205_MOESM6_ESM.pdf]

miRNA	Forward primer (5'-3')	Reverse primer (5'-3')
ssa-miR-135a-3p	GCGCAGTATAGGGATGGA	GTTTTTTTTTTTTTTTGCATGGCT
ssa-miR-135a-5p	CGCAGTATGGCTTTTTATTCCT	GGTCCAGTTTTTTTTTTTTTTTCAGA
ssa-miR-155	CGCAGTTAATGCTAATCGTGATAG	AGGTCCAGTTTTTTTTTTTTTTTACC
ssa-miR-181a-4-3p	ACCATCGACCGTTGAGT	TCCAGTTTTTTTTTTTTTTTGGTACA
ssa-miR-2184-3p	CAGGCACGTAGGCTCT	TCCAGTTTTTTTTTTTTTTTGTACTGT
ssa-miR-20b-5p	AGCAAAGTGCTCACAGTG	GGTCCAGTTTTTTTTTTTTTTTACCT
ssa-let-7a-3p	GCTGTACAGCCTCCTAGC	GGTCCAGTTTTTTTTTTTTTTTGGAA
ssa-miR-125b-3-3p	AGACAGGTGAGGACCTTG	GGTCCAGTTTTTTTTTTTTTTTGTTC
ssa-miR-135b-5p	CGCAGTATGGCTTTCTATTCCT	GGTCCAGTTTTTTTTTTTTTTTCACA
ssa-miR-18a-5p	GCAGTAAGGTGCATCTAGTG	GGTCCAGTTTTTTTTTTTTTTTAACTAC
ssa-miR-216a-5p	GCAGTAATCTCTGCAGGCA	TCCAGTTTTTTTTTTTTTTTCACAGT
n7a-3p	CAGCCAGTATGGCTTGTG	GGTCCAGTTTTTTTTTTTTTTTGTTAG
n7b-3p	CAGCCAGTATGGCTTGTG	CAGTTTTTTTTTTTTTTTGGAGCAG
ssa-miR-1338-5p	CAGAGGACTGTCCAACCT	GGTCCAGTTTTTTTTTTTTTTTATTCTCA
ssa-miR-202-5p	AGAAAGAGGCATAGGGCAT	GTCCAGTTTTTTTTTTTTTTTCCCA
Ssa-miR-202-3p	CGCAGTTCCTATGCATATACC	TCCAGTTTTTTTTTTTTTTTCAAAGC
ssa-miR-222a-3p	CTACATCTGGCTACTGGGT	GGTCCAGTTTTTTTTTTTTTTTGAG
ssa-miR-137-3p	GCAGTTATTGCTTGAGAATACG	CAGGTCCAGTTTTTTTTTTTTTTTCTA
ssa-miR-103-3p	GCAGAGCAGCATTGTACAG	GGTCCAGTTTTTTTTTTTTTTTCATAG
ssa-miR-103-5p	GCAGAGCCTCTTTATGATGCT	CAGTTTTTTTTTTTTTTTACAAGGCA
ssa-miR-25-5p	GCAGAGGCGGAGTCTTG	GTCCAGTTTTTTTTTTTTTTTGGC
